# T_2_ contrast variation in human brain at 7 T and its potential contributors

**DOI:** 10.1162/IMAG.a.67

**Published:** 2025-07-07

**Authors:** Yicun Wang, Peter van Gelderen, Maxime Donadieu, Jiaen Liu, Jacco A. de Zwart, Jiazheng Zhou, Govind Nair, Daniel S. Reich, Jeff H. Duyn

**Affiliations:** Advanced MRI Section, Laboratory of Functional and Molecular Imaging, National Institute of Neurological Disorders and Stroke, National Institutes of Health, Bethesda, MD, United States; Department of Radiology, Renaissance School of Medicine, Stony Brook University, Stony Brook, NY, United States; Translational Neuroradiology Section, National Institute of Neurological Disorders and Stroke, National Institutes of Health, Bethesda, MD, United States; Advanced Imaging Research Center, UT Southwestern Medical Center, Dallas, TX, United States

**Keywords:** brain T_2_ relaxation, high-field MRI, susceptibility imaging, white matter microstructure, orientation dependence

## Abstract

Magnetic susceptibility-weighted MRI (or T_2_^*^-weighted MRI) at 7 T and higher field strengths has shown superb sensitivity to study normal and pathological levels of non-heme (tissue) iron and myelin in the brain. However, macroscopic field perturbations originating from venous vasculature and tissue-air interfaces lead to image artifacts, posing strong confounds to the interpretation of T_2_^*^ contrast. Use of T_2_-based rather than the more common T_2_^*^-based contrast to study susceptibility perturbations may alleviate these adverse effects, but it is technically challenging at high fields. The latter relates to the difficulty in performing accurate RF refocusing in the presence of increased B_0_- and B_1_-non-uniformity, and limits on RF power deposition. To overcome this, we employed the Gradient Echo Sampling of Spin Echo (GESSE) method to study R_2_ (=1/T_2_) variations at 7 T in healthy human brain. Our results indicate that sensitivity of R_2_ to tissue iron, and associated tissue contrast, is largely preserved across subcortical structures, cortical functional areas, and between the cortex and superficial white matter, with substantially reduced sensitivity to macroscopic susceptibility effects. Therefore, R_2_ as measured by GESSE may complement current R_2_^*^- and χ-based approaches for quantification of brain tissue iron and myelin. In deep white matter, R_2_ was found to exhibit fiber bundle specificity, and showed significant correlations with documented fiber diameter and inferred orientation dependence with respect to the B_0_. These results comprehensively chart multiple main contributors to R_2_ contrast at 7 T across the whole brain, extending previous studies that have done so in specific brain areas or at lower field. Quantitative interpretation of R_2_ contrast in terms of tissue iron and myelin content needs to take all these contributors into account.

## 1 Introduction

The development of high-field MRI over the last two decades has been motivated by the promise of increased sensitivity and contrast for clinical neuroimaging. While high-field MRI has been beneficial for several applications, particularly significant improvements have been obtained with the use of T_2_^*^-weighted techniques that are sensitive to tissue magnetic susceptibility χ. These techniques are sensitive to subtle variations in χ associated with the presence of non-heme tissue iron and myelin, brain constituents relevant for healthy human brain function. Based on this, magnetic susceptibility contrast at 7 T has been used to study tissue iron accumulation in healthy aging (Betts et al., 2016; Buijs et al., 2017), Alzheimer’s disease (McKiernan & O’Brien, 2017), and Parkinson’s disease (Lehéricy et al., 2014; Schwarz et al., 2018). In multiple sclerosis, both white matter and cortical demyelinating lesions can be detected at 7 T (Liu et al., 2021; Nielsen et al., 2013), as well as aberrant iron deposition in and around the lesions that are associated with neuroinflammation (Absinta et al., 2016; Bagnato et al., 2011). This strong sensitivity provided by 7 T MRI offers opportunities to quantify local changes of tissue iron and myelin (Shin et al., 2021; Z. Li et al., 2023), potentially assisting diagnosis and monitoring of brain conditions and therapeutic interventions. In favor of readability, we will refer to non-heme tissue iron simply as iron in the following, noting the differences in relaxometry between non-heme and heme iron (Yablonskiy et al., 2021).

One of the outstanding problems with susceptibility contrast in MRI is its sensitivity to image artifacts around strong magnetic perturbations (e.g., from veins and air/tissue interfaces), which can compromise image quality and confound interpretation. These adverse effects can be further complicated by temporal field changes during the scan due to respiration and head motion (Liu et al., 2018; van Gelderen et al., 2007). In addition, quantitative evaluation of iron and myelin is affected by anisotropic microstructure (e.g., sensitivity to the orientation of white matter fibers relative to B_0_) (Lee et al., 2010), an issue remaining incompletely resolved. Inclusion of fiber orientation data (e.g., from diffusion MRI) may help address this problem but requires extra scan time and novel reconstruction techniques (X. Li et al., 2012) that have not yet reached clinical standards.

An alternative approach to study brain iron and myelin is the use of T_2_ contrast, which is less affected by artifacts from veins and air/tissue interfaces while maintaining sensitivity to iron (Drayer et al., 1986; Hasan et al., 2012; Langkammer et al., 2010; Vymazal et al., 1999) and myelin (Cho et al., 2023; Leppert et al., 2009; Miot-Noirault et al., 1997), albeit to a lesser degree than T_2_^*^. Like with T_2_^*^, the sensitivity of T_2_ to tissue iron has been shown to increase with magnetic field at least up to 7 T (Bartzokis et al., 1993; Mitsumori et al., 2012; Schenck, 1995; van Gelderen et al., 2025). Exploiting the sensitivity of T_2_ to iron for iron quantification is complicated by potential contributions of myelin to T_2_ (van Gelderen et al., 2025). The sensitivity of T_2_ to myelin has not been well explored at 7 T and above, and neither has the potential effect of white matter fiber microstructure and orientation on T_2_. In part, this paucity of T_2_ research relates to the difficulty in performing quantitative T_2_ measurements at ultra-high-field, arising from increased RF power deposition and B_0_- and B_1_ nonuniformity. The latter compromise the ability to achieve accurate RF refocusing in spin echo (SE) generation required for sensitization to T_2_.

Some of the above challenges with established approaches to measure T_2_ may be alleviated by employing a method called Gradient Echo Sampling of Spin Echo (GESSE) (Cox & Gowland, 2010; Ma & Wehrli, 1996; Yablonskiy & Haacke, 1997), in which a single SE is generated and sampled with multiple gradient echoes (GEs). From an imaging practicality perspective, GESSE does not suffer from accumulating refocusing imperfections of multi-SE acquisitions, or time-inefficient measurement of T_2_ with multiple single SE acquisitions at varying spin echo TE (sTE). These technical features make GESSE a more feasible approach for T_2_ mapping at ultra-high-field, despite expected discrepancies in the obtained T_2_ value when compared to that derived from conventional CPMG and Hahn-echo methods.

The goals of this study were to employ GESSE at 7 T to explore quantitative T_2_ measures across the brain in both gray and white matter, and to investigate potential influences of white matter microstructure and orientation on measured T_2_ values.

## 2 Methods

### 2.1 Overview

**Fig. 1.**
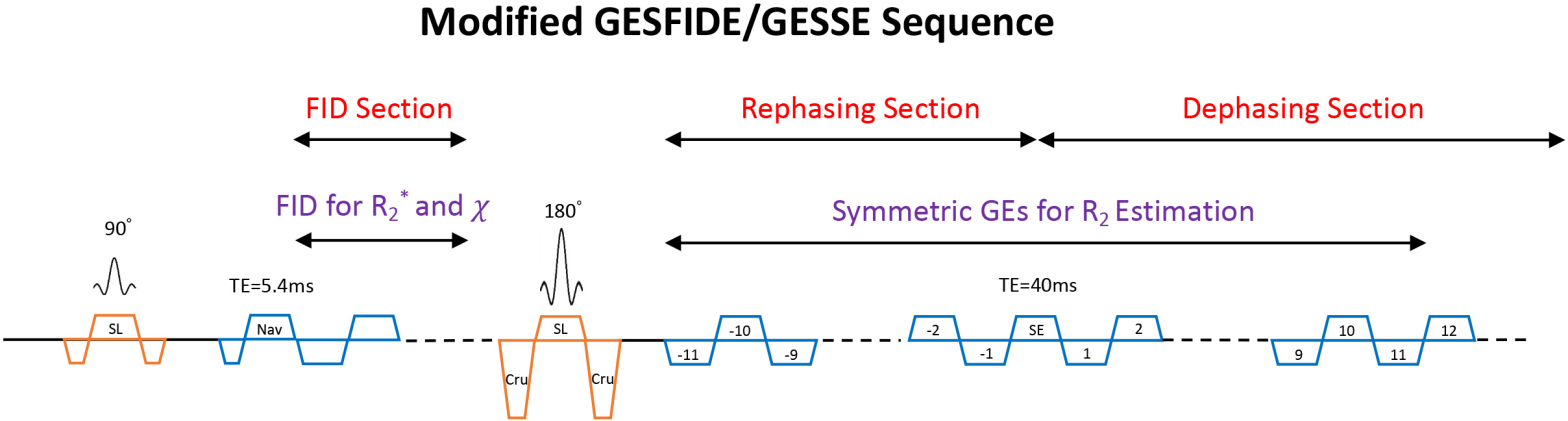
Schematic of the modified GESFIDE/GESSE sequence. Gradient echoes are grouped into 3 sections: FID, rephasing, and dephasing. SL, slice selective gradient; Nav, navigator echo; Cru, crusher; SE, spin echo. Positive and negative echo numbers denote symmetric gradient echo pairs about the SE for R_2_ estimation based on the GESSE method. R_2_^*^ and χ were computed using data from the FID section. Phase encoding gradients and end-of-TR crushers are omitted for simplicity.

We implemented a modified GESFIDE/GESSE sequence that samples the Free Induction Decay (FID), as well as the dephasing and rephasing sides of the SE (Ni et al., 2015) (Fig. 1). The GE samples around the SE were used to estimate R_2_ as proposed by the original GESSE study (Yablonskiy & Haacke, 1997) to take advantage of its features described above. The FID section was used to estimate R_2_^*^ and χ, yielding quantitative images on par with those from commonly used GRE sequences with short TEs.

After first implementing and optimizing the acquisition protocol at 7 T to enable whole-brain coverage, we performed R_2_ (=1/T_2_) analysis on a group of healthy volunteers. The latter was done using both surface- and volume-based analyses. Initial inspection of R_2_ within the white matter revealed strong variation that appeared to be fiber tract-specific, indicating potential dependence of fiber diameter or fiber orientation in the main magnetic field (B_0_). To investigate the effects of fiber diameter, we conducted sectional analysis of R_2_ on the corpus callosum in sagittal images where fibers in each section were uniformly oriented perpendicular to B_0_ but had varying diameters (Aboitiz et al., 1992). To investigate the contribution of fiber orientation, we performed voxel-wise fiber orientation calculation and fiber tract segmentation, followed by joint regression of the measured R_2_ values on fiber orientation and diffusion-based fiber diameter index reported in S. Y. Huang et al. (2020).

### 2.2 MRI data acquisition

The modified GESFIDE/GESSE sequence was implemented on a clinical 7 T MRI system (Terra, Siemens Healthineers, Erlangen, Germany) equipped with a single-channel transmit, 32-channel receive head RF coil array (Nova Medical, Wilmington, USA). In vivo experiments were conducted under a human subject research protocol approved by the Institutional Review Board at the National Institutes of Health.

Twelve healthy volunteers (4 males, 23–34 years) were scanned. Images were acquired from transverse-oblique slices parallel to the AC-PC line, with FOV 240 × 180 mm^2^, resolution 1.0 × 1.0 mm^2^, slice thickness 2 mm, slice gap 1 mm, 36–38 slices covering the entire brain, SE formation at sTE 40 ms, echo spacing (ΔTE ) 1.30 ms, bandwidth 947 Hz/voxel, 6 GEs in the FID section (echo train TE 7.0–13.5 ms), 11 GEs in the rephasing section (echo train TE 25.7–40.0 ms), and 30 GEs in the dephasing section (echo train TE 40.0–79.0 ms). A tapered-sinc pulse with a time-bandwidth product of 6.0 was used for both excitation and refocusing. A slice refocusing factor of 1.5 (thickness ratio of refocused over excited slices) was used to alleviate the issue of imperfectly refocused slice profile. This was achieved by increasing the bandwidth of the refocusing pulse by a factor of 1.5, such that the slice-selection gradient remained the same for both pulses. This had the advantage of consistency between the excited and refocused slice positions in the presence of B_0_ inhomogeneity. The refocusing RF pulse had a duration of 5 ms, constrained by limits on peak RF amplitude and power deposition (“SAR limit”). The excitation RF pulse length was 7.5 ms. TR was varied from 4.5–5.5 s to comply with the scanner prescribed “normal-mode” SAR limit (3.2 W/kg). A first-order navigator was acquired at TE = 5.4 ms to correct for the frequency fluctuations by instrumental and physiological sources. The scan time was approximately 15 min, which was achieved without the use of parallel imaging or partial Fourier acceleration. On subsequent GEs, there were small alternating image distortions along the readout direction corresponding to the positive-negative polarities of the odd/even numbered readout gradients in the presence of B_0_ inhomogeneity, especially above the nasal sinus. These were corrected based on known readout gradient strength and B_0_ field variations estimated by comparing images from a pair of subsequent GEs around the SE. Additionally, data with higher signal-to-noise ratio were acquired by collecting shorter scans (TR = 2 s) which were averaged over 3–4 repetitions. These data were acquired as transverse-oblique slices in 2 subjects for demonstration purposes, and sagittal images in 6 subjects were acquired to visualize the corpus callosum and the corticospinal tract.

A 3D T_1_-MP2RAGE with 0.75 mm isotropic resolution was acquired as anatomical reference using the following parameters: 4600 ms TR, 840 ms TI1, 2370 ms TI2, and 2.3 ms TE. Flip angle was 5^°^ and 6^°^ for the 2 TIs respectively. The total acquisition time was 10 min.

### 2.3 Data analysis

#### 2.3.1 R_2_ calculation

The GESSE signal equation is as follows (Cox & Gowland, 2010; Yablonskiy & Haacke, 1997):

$$\begin{array}{l} S(TE)=M_{0}\text{exp}(-R_{2}\cdot TE-{R^{\prime }}_{2}\cdot \left|TE-sTE\right|)\\ \;\;\;\;\;\;\;\;\;\;\;\;\;\;\;\;=M_{0}\text{exp}(-R_{2}\cdot TE-{R^{\prime }}_{2}\cdot n\Delta TE) \end{array}$$

where S denotes image magnitude, *$M_{0}$* is steady-state signal magnitude, ΔTE echo spacing, and n the distance from sTE in units of ΔTE . GE pairs symmetric around sTE = 40 ms were used to calculate R_2_ maps as follows

$$R_{2}(n\Delta TE)=\text{ln}[S(sTE-n\Delta TE)\;/\;S(sTE+n\Delta TE)]\;/\;(2n\Delta TE)$$

Empirically, it was found that R_2_ maps corresponding to n = 6–11 (7.0–12.8 ms away from the SE top) had sufficiently high CNR to be averaged for further processing (Supplementary Fig. S1). $R_{2}$ derived from this method (averaging of normalized ratios) was compared to exponential fitting to the signal S, as well as linear fitting to the above function, both in the sense of least squared errors. Similar goodness of fit and R_2_ values were obtained using these methods across representative tissue types (Supplementary Figs. S2 and S3). Therefore, we opted for the simple and fast “model-free” approach that does not require pixel-wise fitting.

#### 2.3.2 Calculation of T_2_^*^ and χ

T_2_^*^ and QSM χ maps were computed with JHU/KKI QSM toolbox v3.3 (https://github.com/xuli99/JHUKKI_QSM_Toolbox) (Bao et al., 2016; X. Li et al., 2019; van Bergen et al., 2016) using the FID data after the excitation pulse. Processing steps include phase unwrapping, brain extraction using BET in FSL (Smith, 2002), background field removal using VSHARP (kernel size 10 mm) (Wu et al., 2012), and dipole inversion using a modified structural feature collaborative reconstruction method (Bao et al., 2016). T_2_^*^ and χ maps were intrinsically registered to T_2_ as they were from the same acquisition.

#### 2.3.3 Cortical surface analysis

FreeSurfer (v7.2.0, https://surfer.nmr.mgh.harvard.edu/) was used to generate cortical surfaces and volumetric regions of interests (ROIs) using the “recon-all” command on the T_1_-MP2RAGE. GE images at the TE (gTE) of 30 ms were affinely registered to the 3D T_1_-MP2RAGE reference image set, yielding a registration matrix which was used to transfer data from the GE image space to the reference space (to generate R_2_ cortical surface) and vice versa (to obtain ROI labels in the original R_2_ space). For each subject, R_2_ was averaged across 25% to 75% cortical depth to obtain a cortical surface map, and across -25% to -75% cortical depth to obtain a superficial white matter (SWM) map (i.e., white matter immediately below the cortical ribbon). The surface maps were topographically normalized to the “fsaverage” template for group averaging. Volumetric ROIs were taken from the “aparc+aseg.mgz” and “wmparc.mgz” output in the T_1_-MP2RAGE reference space, including cortical functional areas, white matter areas within four brain lobes, as well as some subcortical nuclei. To alleviate partial volume effects, each binary ROI mask was tri-linearly transformed to the R_2_ space separately, and a threshold of 0.9 was applied to re-generate a binary mask. The red nuclei and substantia nigra were not included in the segmentation; therefore, they were manually drawn for each subject on the GE images.

#### 2.3.4 Comparison of R_2_ and effective fiber diameter across corpus callosum sections

R_2_ of the corpus callosum was analyzed on the central 3 sagittal slices located around the interhemispheric fissure, corresponding to a lateral width of 20 mm (2 mm slice thickness and 7 mm gap), in which fiber tracts are predominantly perpendicular to the B_0_ field. The corpus callosum was manually segmented and divided from tip to tip into four equal-length sections. From anterior to posterior, these sections are genu, anterior body, isthmus, and splenium.

Effective fiber diameter was defined as the average fiber diameter weighted by the corresponding cross-sectional area, that is,

$$d_{eff}=\frac{\sum_{i}d_{i}\cdot f_{i}\cdot \pi {\left(d_{i}\;/2\right)}^{2}}{\sum_{i}f_{i}\cdot \pi {\left(d_{i}\;/\;2\right)}^{2}}=\frac{\sum_{i}f_{i}d_{i}^{3}}{\sum_{i}f_{i}d_{i}^{2}}$$

where $d_{i}$ and $f_{i}$ are fiber diameter and frequency extracted from the histograms reported in Aboitiz et al. (1992, figure 4). Data from “anterior body” and “mid body” in that figure were combined into a single section of anterior body in this study, due to the limited number of MRI voxels in this narrow region of the corpus callosum.

#### 2.3.5 White matter tract analysis

To facilitate comparison of white matter fiber tract R_2_ in this study with diffusion MRI-derived fiber diameter index reported by S. Y. Huang et al. (2020), we generated the same 20 fiber tract ROIs by implementing a similar post-processing pipeline based on NiftyReg (http://cmictig.cs.ucl.ac.uk/wiki/index.php/NiftyReg). First, a 3D GE volume (gTE = 30 ms) was registered to the T_2_-weighted template in MNI-152 space from the Johns Hopkins University (JHU) white-matter probabilistic tractography atlas (Mori et al., 2008), using linear registration (“reg_aladin”) followed by nonlinear warping (“reg_f3d”). Then, the transformation was inverted to obtain the probabilistic tractography map in the subject native space for each fiber tract. A threshold of 0.25 was taken to generate binary ROIs. In the voxel-wise analysis, the apparent axonal diameter (d) for each individual GESSE voxel was set to the average diameter from (S. Y. Huang et al., 2020) for the fiber tract to which the voxel was assigned based on the registration to the JHU atlas.

To study the effect of fiber orientation on R_2_, the same inverse transformation was applied to the fractional anisotropy (FA) map and principal eigenvector (PEV) images in the same JHU atlas. The PEV map was then combined with the slice orientation (axial or axial oblique) to calculate the angle α between the primary fiber orientation and the B_0_ field. White matter voxels in the native space with an FA larger than 0.4 and tract probability higher than 0.25 were included in this analysis, resulting in 49,967 valid voxels from the 12 subjects. Finally, $sin^{4}\alpha$ was calculated for each voxel to describe the orientation dependence of R_2_ (Bartels et al., 2022; Gil et al., 2016; Knight et al., 2017). Voxels for each fiber tract were categorized into 10 bins based on their $sin^{4}\alpha$ values (ranging from 0 to 1) with a bin width of 0.1. The fitting described below was performed on the binned data to alleviate the heavy weighting on the fibers that were close to being aligned with or perpendicular to B_0_ (Supplementary Fig. S4), a consequence of the natural head pose in supine position.

Multiple linear regression was performed using the following model:

$$R_{2}=a_{0}+a_{1}(d-\bar{d})+a_{2}sin^{4}\alpha +a_{3}(d-\bar{d})sin^{4}\alpha$$

where $a_{0,1,2,3}$ are fitting coefficients, d denotes the apparent axonal diameter (mean value over voxels belonging to the same fiber tract, as described above for d), and $\bar{d}$ is the mean diameter of all fiber tracts weighted by their corresponding number of voxels (calculated to be 4.46 μm). Using this “de-meaned” term of $\left(d-\bar{d}\right)$ instead of d helps to improve the interpretability of the intercept $a_{0}$, which represents the R_2_ of a white matter voxel of average axonal diameter $\left(d-\bar{d}=0\right)$ and with its fiber tract aligned with B_0_ ($sin^{4}\alpha =0$ ). A subset of the coefficients was set to 0 to investigate the explanatory power of the remaining terms using the R^2^ and adjusted R^2^ metrics.

## 3 Results

### 3.1 Overview

**Fig. 2.**
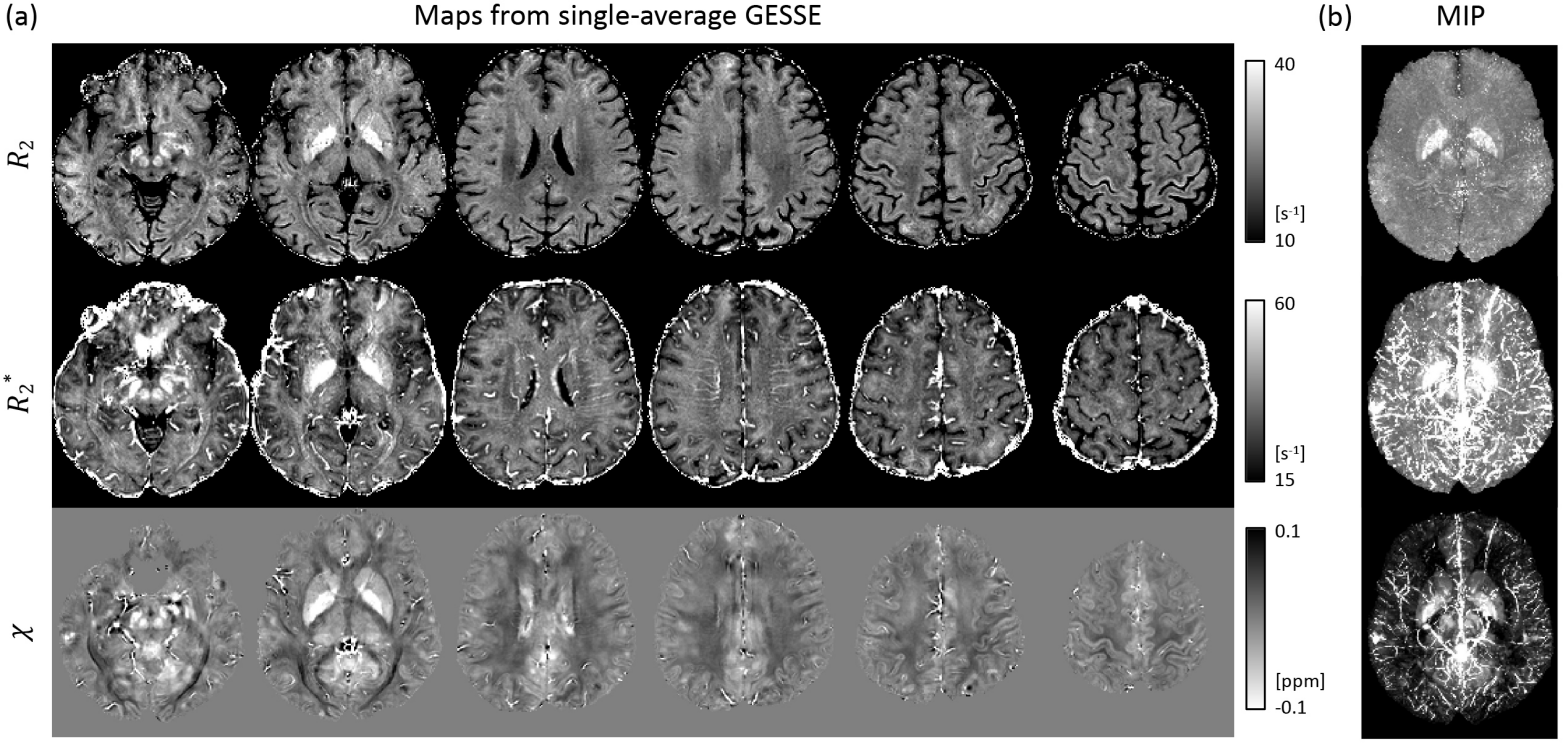
R_2_, R_2_^*^ and χ derived from a single modified GESFIDE/GESSE scan (a). Shown are representative slices from 36 slices that covered the whole brain. MIP, maximum intensity projection across slices (b).

Example R_2_, R_2_^*^, and χ maps derived from a single modified GESFIDE/GESSE scan at 7 T are shown in Figure 2a. While iron-rich subcortical structures (e.g., basal ganglia, substantial nigra, red nucleus, and the pulvinar of thalamus) are conspicuous in all three contrasts, these maps show several notable differences. First, compared to R_2_^*^ and χ, R_2_ is much less sensitive to the venous vasculature, as observed most prominently on the maximum intensity projection (MIP) images across slices (Fig. 2b). This is attributed to the fact that the spatial scale of susceptibility perturbations introduced by the veins is much larger than the diffusion distance between excitation and sTE (~5–10 μm), leading to effective refocusing (Boxerman et al., 1995; Weisskoff et al., 1994). Second, compared to R_2_^*^, stronger R_2_ variation across the cortex can be observed, as well as stronger changes of the contrast between cortical gray matter versus superficial white matter. Third, within the deep white matter, there are sharp contrast transitions that appear to be fiber-tract specific. In the following, we study these features in more detail.

### 3.2 Cortical and subcortical contrast variations

**Fig. 3.**
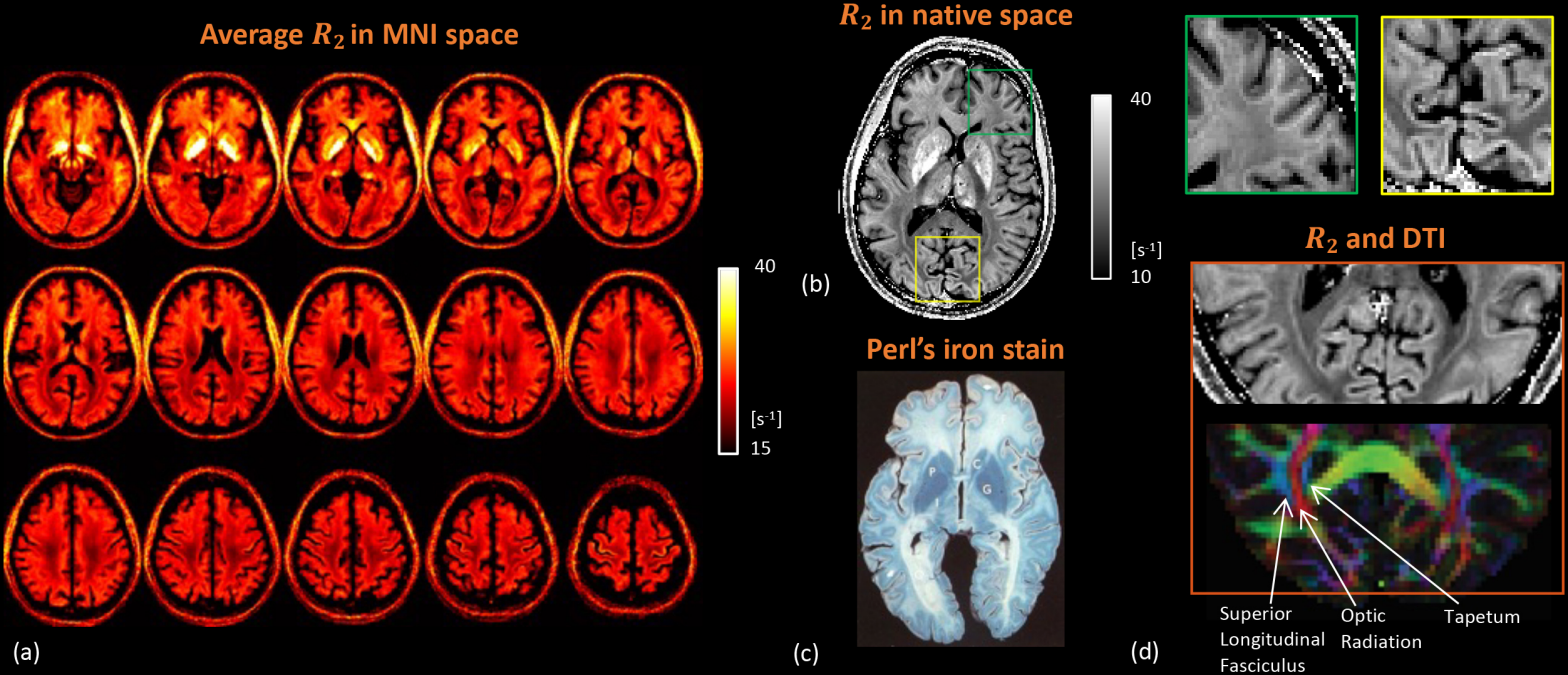
Colocalization of high R_2_ at 7 T with iron-rich structures and certain white matter bundles. Group-averaged R_2_ in normalized MNI space exhibiting topographical variations across the whole brain (a). Single slice R_2_ with 4 averages (b) compared to Perl’s iron stain in Drayer et al. (1986), reprinted with permission (c). The zoomed area in the green box shows frontal superficial white matter, the yellow box shows occipital cortex. In both cases, the annotated structure has higher R_2_ than the adjacent tissue. In deep white matter, the optic radiation has higher R_2_ than adjacent superior longitudinal fasciculus and tapetum (d). DTI (Diffusion Tensor Imaging) data acquired from a different healthy volunteer at 3 T for demonstration purpose.

**Fig. 4.**
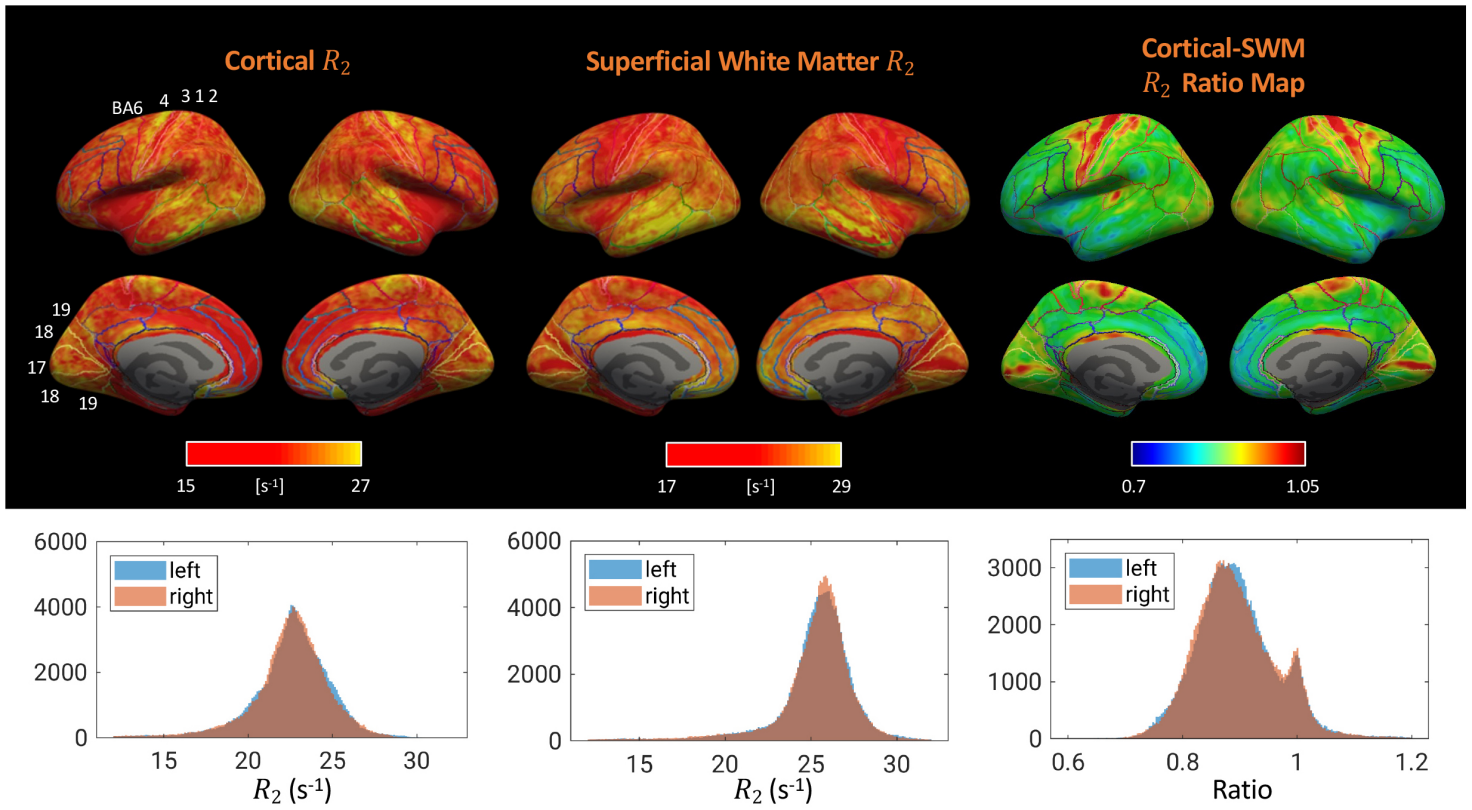
R_2_ on the cortical surface, superficial white matter surface, their ratio, and corresponding histograms for both left and right hemispheres. Labeled areas are 6 premotor, 4 primary motor, 3/1/2 primary somatosensory, 17 primary visual, 18 secondary visual, and 19 associative visual. Results from a group of healthy subjects, n = 12. The color bars (display ranges) for the surface maps were rectified, that is, not corresponding to the minimum and maximum of the underlying data as shown in the histograms to improve visualization of the overall pattern.

**Fig. 5.**
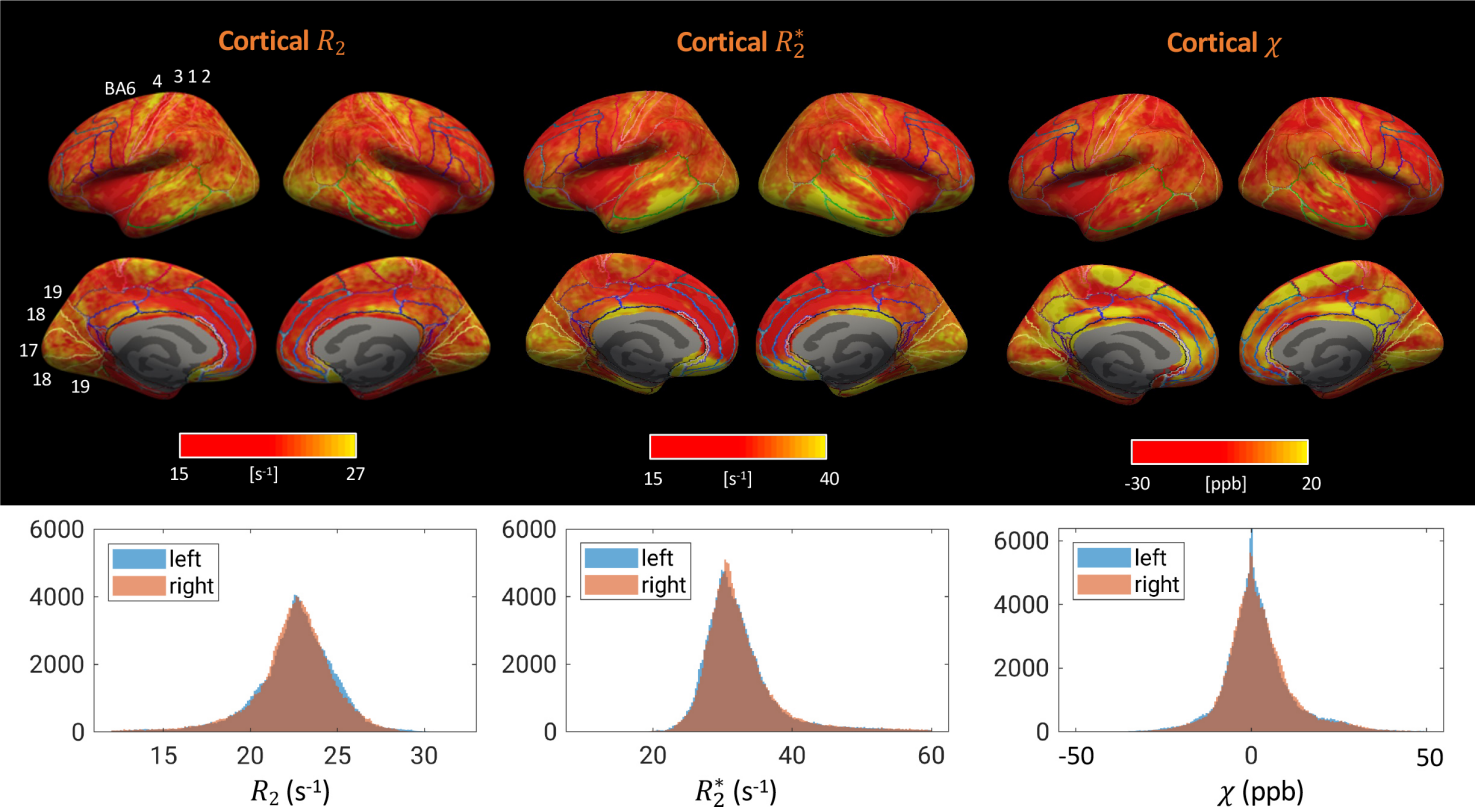
R_2_ (same as in Fig. 4), R_2_^*^ and χ maps on the cortical surface, and corresponding histograms for both left and right hemispheres. Colored lines denote Brodmann cytoarchitectural areas (BA). Labeled areas are 6 premotor, 4 primary motor, 3/1/2 primary somatosensory, 17 primary visual, 18 secondary visual, and 19 associative visual. Results from a group of healthy subjects, n = 12. The color bars were rectified as described in the caption of Figure 4.

As is apparent from Figure 2, substantial R_2_ variations are seen in both cortex and the underlying (superficial) white matter. This has been previously observed at 1.5 T, and tentatively attributed to variations in iron content (Zhou et al., 2001). The nature of these R_2_ variations is further explored in Figures 3 to 5. Across the brain, contrast between cortical gray and superficial white matter is highly variable: in much of the frontal, temporal lobes and the insula, superficial white matter R_2_ exceeds cortical R_2_, while the reverse is true in the occipital and parietal lobes (Fig. 3a). A notable exception in the frontal lobe is the motor cortex R_2_, where gray matter R_2_ exceeds white matter R_2_ (Fig. 3a). At the higher resolution of individual (native space) data, it appears that in the frontal and temporal lobes, a thin strip of high R_2_ tissue closely follows the gray-white matter border (Fig. 3b), presumably originating from the thin layer of iron-containing U-fibers in the superficial white matter (Kirilina et al., 2020). This is also apparent on a previously published Perl’s iron stain (Fig. 3c).

The spatial variation of gray-white matter R_2_ contrast is more evident when viewed from the cortical surface (Fig. 4). Cortical gray and superficial white matter R_2_ generally follow Brodmann areas (BA) parcellations and display complementary patterns. Primary cortical areas such as the primary motor (BA 4), somatosensory (BA 3, 1, 2), and visual (BA 17) areas have higher R_2_, potentially due to higher non-heme iron content. R_2_ in the superficial white matter is lower, in contrast to that in the frontal and temporal lobes. These observations are consistent with, and nicely complemented by a recent study using high-resolution (250 μm) line-scanning GESSE at 7 T that reports higher R_2_ in the primary visual, primary motor and somatosensory cortices, compared to adjacent white matter (Balasubramanian et al., 2022). In addition, superficial white matter R_2_ in the occipital pole manifests clear correspondence to visual subareas, with the associative visual area (BA 18) having higher R_2_ than its neighbors, the primary visual (BA 17) and associative visual (BA 19) areas. When taking the ratio of cortical and superficial white matter surfaces, a high-contrast map yields that is specific to functional areas, highlighting the sensorimotor, primary visual, primary auditory, and cingulate cortices. This resulted in an intriguing bi-modal distribution of the ratio values clearly seen on its histogram, which was absent for both cortical and SWM R_2_. All surface maps are highly symmetric between left and right hemispheres.

In comparison to R_2_, cortical susceptibility maps based on R_2_^*^ and χ are more strongly impacted by tissue-air interfaces and the venous vasculature (Fig. 5): The exceedingly high R_2_^*^ values in the lower temporal and lower frontal lobes are due to their vicinity to the ear canals and nasal sinus, respectively, consistent with a previous report on cortical R_2_^*^ at 7 T (Cohen-Adad et al., 2012). The high χ areas are adjacent to the superior and inferior sagittal sinuses on the mid-sagittal plane, likely being results of incomplete dipole inversion near those strong susceptibility sources. These are consistent with the observations made in Figure 2.

**Fig. 6.**
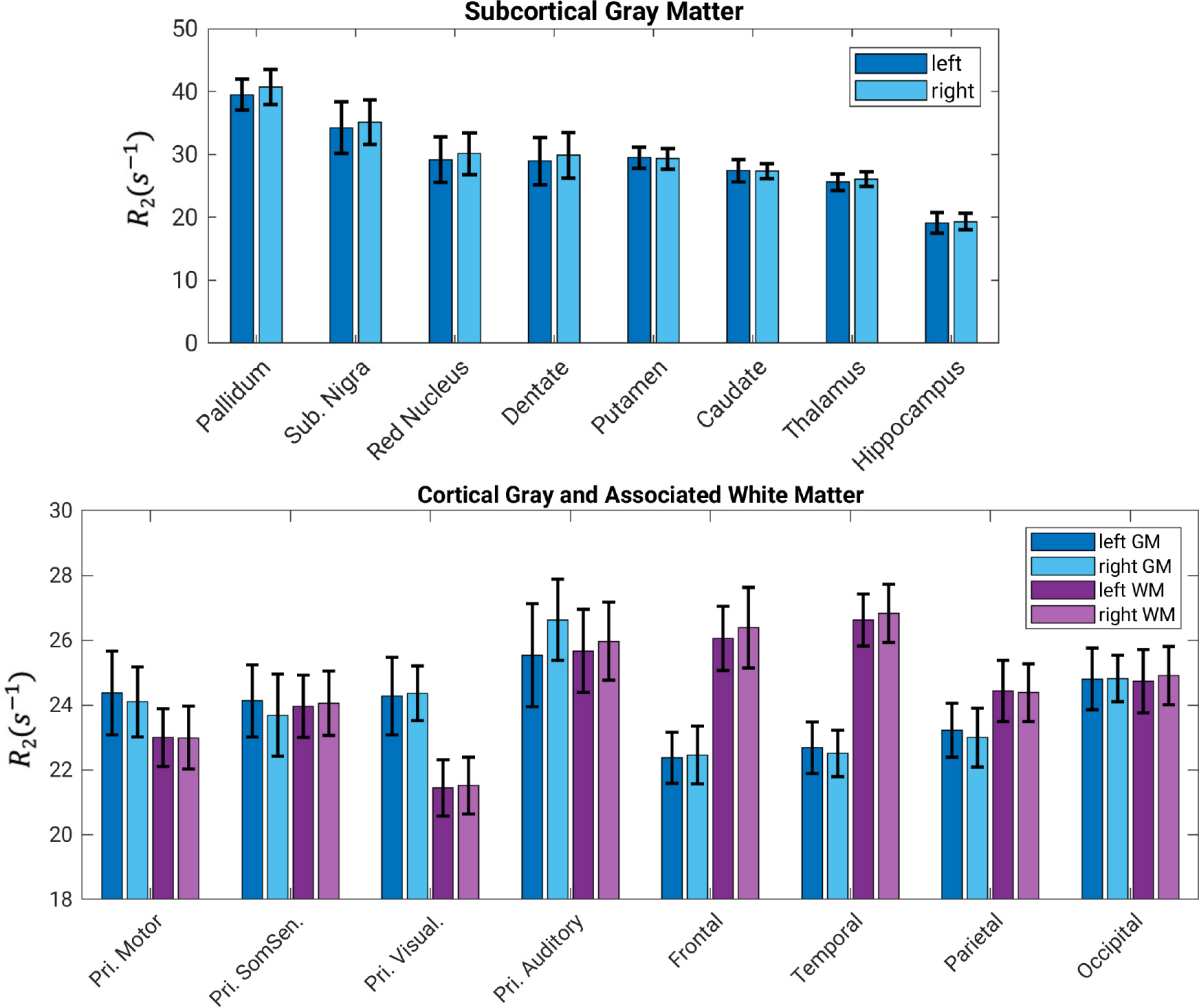
Statistics of R_2_ in subcortical structures, cortex and associated white matter areas. Data are shown as mean ± standard deviation over 12 young volunteers, for both left and right hemispheres. Results for cortical areas by lobes exclude primary motor, primary somatosensory, primary visual and primary auditory cortices, which are listed separately.

R_2_ results from volume and surface ROIs are summarized in Figure 6. Distinct from R_1_ and R_2_^*^ in which the white matter is typically higher (relaxing faster) than the cortex, R_2_ of white matter and cortical areas at 7 T fluctuate within a similar range of 22-28 s^-1^. The results corroborate our observations that the cortical-white matter R_2_ contrast varies and even reverses across the brain. In particular, cortical R_2_ is higher than the underlying white matter R_2_ for the primary motor and visual cortices, consistent with the results in Figure 4. Nevertheless, such depth dependence was absent from the primary somatosensory and auditory cortices at the group level, which will be discussed below.

### 3.3 White matter tracts

In addition to the high contrast across gray matter, between the cortex and superficial white mater, and between different white matter regions, a strong R_2_ variation of 6 s^-1^, or 25%, can be observed within the white matter, with prominent tract specificity (Figs. 2a and 3a, b). For example, in the occipital lobe, the tapetum and the superior longitudinal fasciculus have notably lower R_2_ than the optic radiation between them (Fig. 3b, enlarged in Fig. 3d). This variation is not readily explained by differences in iron content, as histological iron stains typically suggest low iron levels in most major fiber bundles in deep white matter (Drayer et al., 1986; Fukunaga et al., 2010) (Fig. 3c). This high level of spatial specificity is also absent from this area in myelin stain maps (Hametner et al., 2018). To investigate potential contributions from fiber microstructure, we performed tract-specific analysis.

**Fig. 7.**
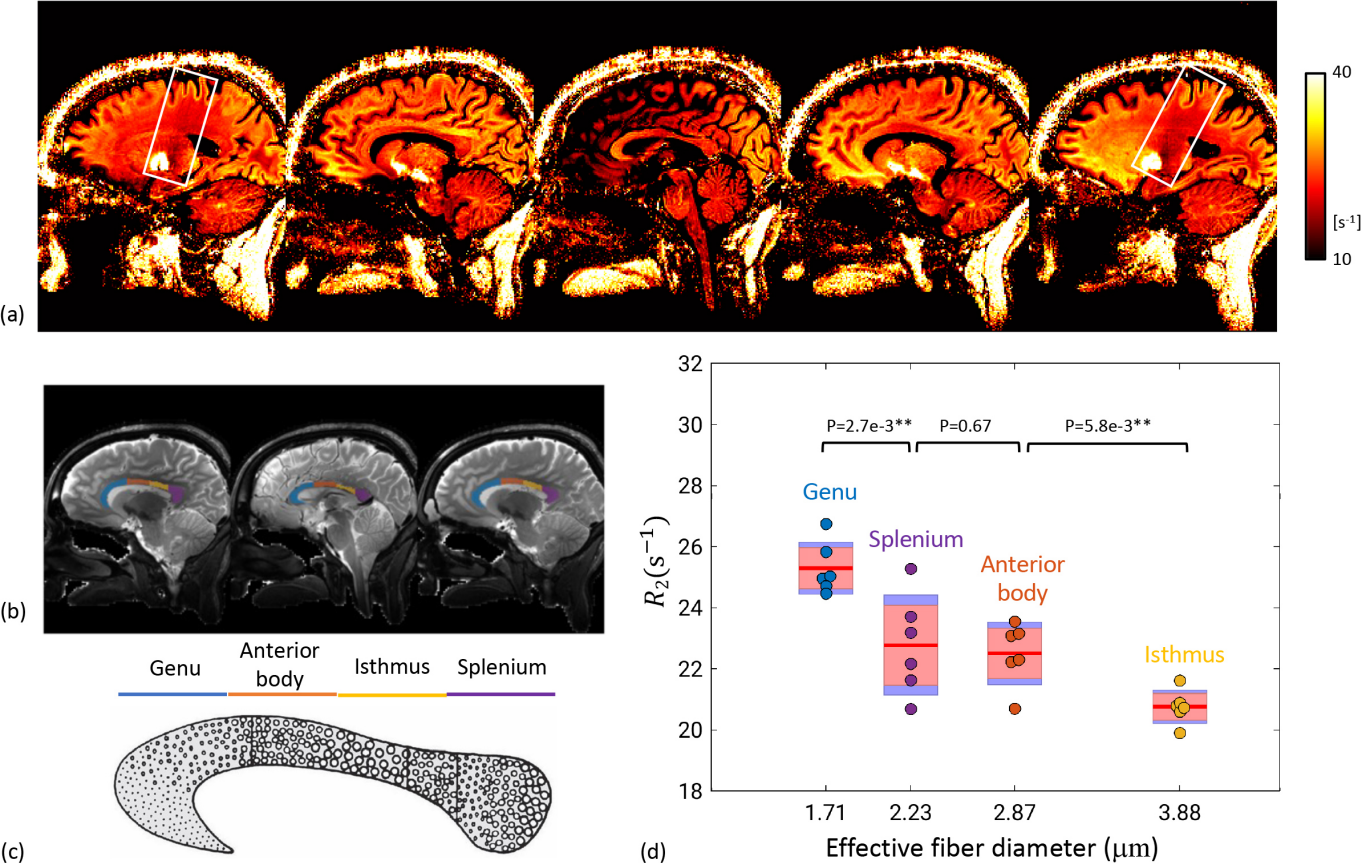
R_2_ map on sagittal slices (a). Images were obtained from 3 averages worth of data. The white boxes frame the bilateral corticospinal tracts that have lower R_2_, connecting the globus pallidus and the motor cortex that have higher R_2_. Example sections of the corpus callosum (b) in comparison with illustration of axon size composition from (Aboitiz & Montiel, 2003), reprinted under the CC BY License without change (c). Their R_2_ statistics over 6 subjects are plotted against effective fiber diameter from electron microscopy (d). Dots denote results from single subjects with the same colors as in the mask; red line is the group mean; red block is 95% confidence interval; blue line denotes 1 standard deviation. **p < 0.001.

We started from the corpus callosum, which consists of multiple fiber tracts traversing between left and right hemispheres. On sagittal images, these fiber tracts are arranged in segments from anterior to posterior with known variation in fiber diameter under electron microscopy (Aboitiz et al., 1992). This variation is associated with the functions these tracts subserve, with fibers connecting primary auditory cortex (in isthmus in Fig. 7c) being the largest and fastest signal carriers. Consistently high R_2_ was found in the fibers of the genu that had smaller diameters, and low R_2_ in the isthmus (Fig. 7a; Supplementary Fig. S5). R_2_ values of the four sections studied showed a trend of inverse correlation with the effective fiber diameter (Fig. 7d). In addition, on the lateral slices, the corticospinal tracts are clearly visible with lower R_2_. The gray matter structures it connects, that is, the globus pallidus and the motor cortex, are also discernable with higher R_2_ (Fig. 7a).

**Fig. 8.**
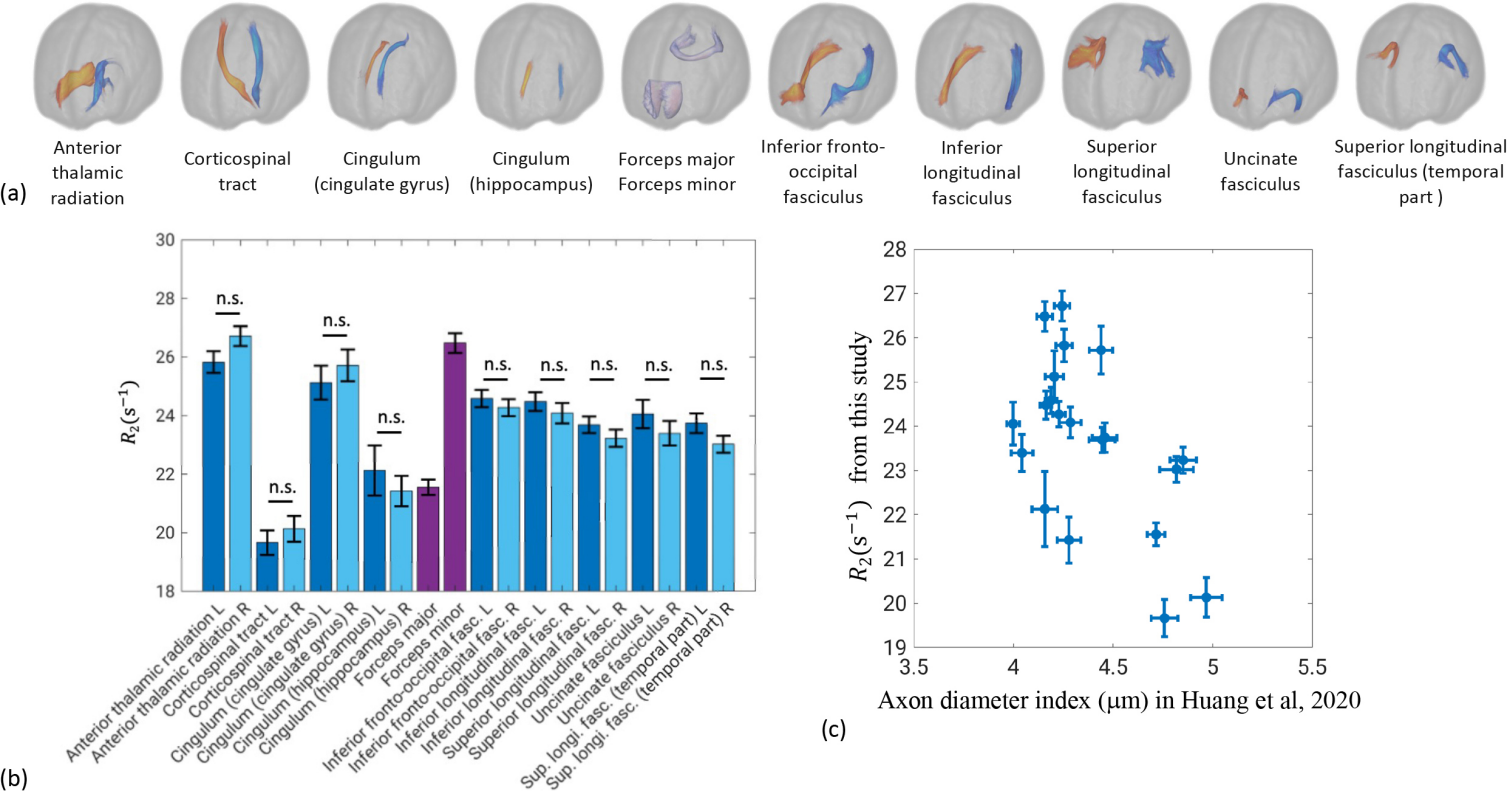
Tract-specific R_2_ (b) in 20 white matter fiber tracts (a, taken from S. Y. Huang et al., 2020). Data are shown as mean ± standard error over 12 subjects. Statistical tests were performed for left-right fiber tract pairs. n.s. not significant (p > 0.05 after correction for multiple comparison). Scatter plot of R_2_ versus diffusion MRI derived axon diameter index for the same tracts in S. Y. Huang et al. (2020) (c). All error bars in standard error.

Back to the whole-brain axial images, statistical analysis of R_2_ within 20 major fiber tracts is shown in Figure 8. The tract-specific R_2_ variation was consistent for left versus right hemispheres. Across white matter tracts, R_2_ manifested a trend of inverse correlation with apparent axonal diameter d from diffusion measures using high-performance gradients (S. Y. Huang et al., 2020). This result is consistent with the findings in the corpus callosum.

**Fig. 9.**
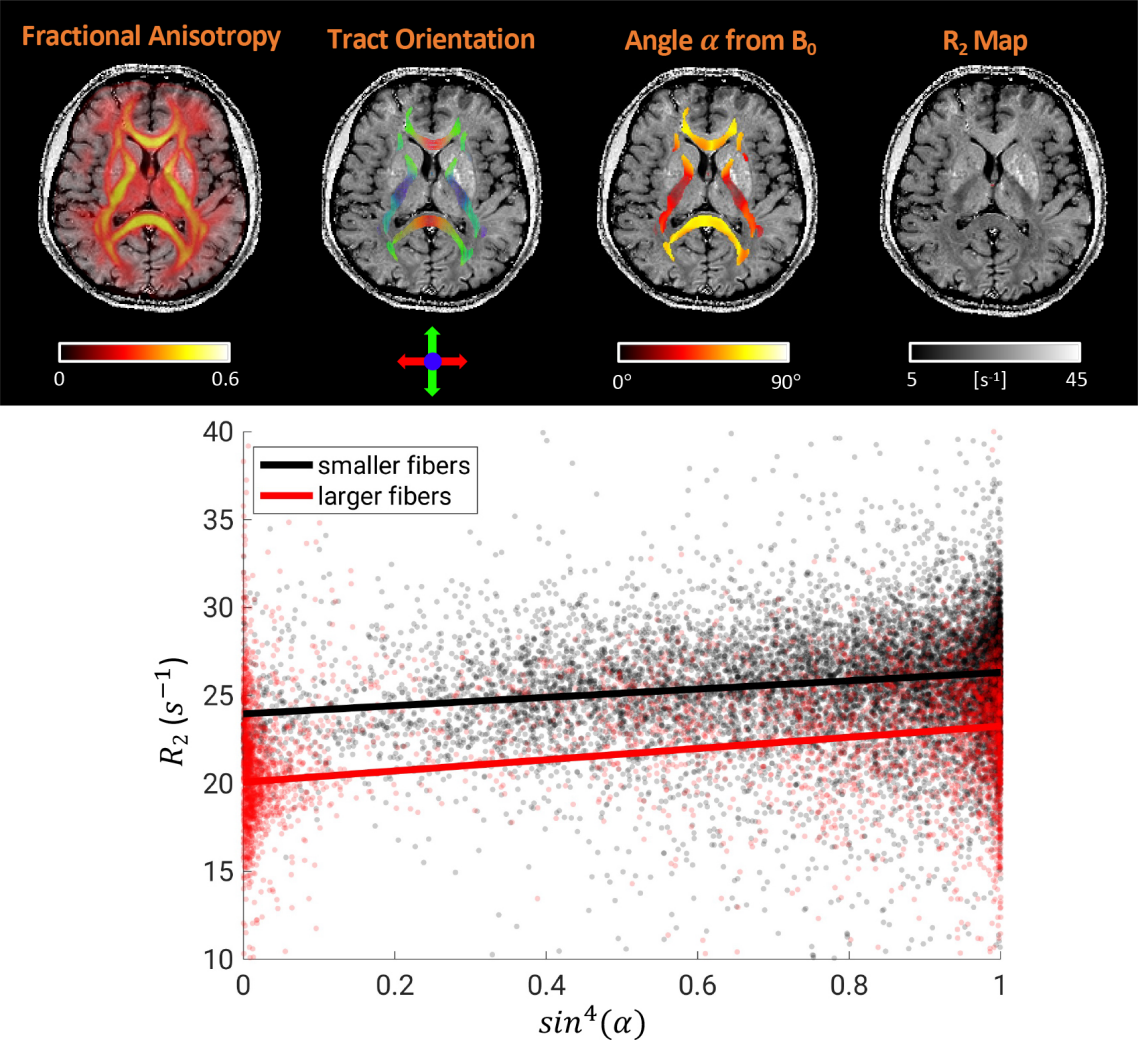
Example calculation for fiber tract orientation (top). Fit of R_2_ against angle function $sin^{4}\alpha$ , separately for fibers with diameter larger (red, $R_{2}=20.08+3.19sin^{4}\alpha$ ) and smaller (black, $R_{2}=23.95+2.36sin^{4}\alpha$ ) than the average (4.46 μm), using data from a single subject (bottom).

Figure 9 shows an example of fiber orientation calculation by registration to the JHU atlas, as an angle α (range 0^°^–90^°^) with respect to the B_0_. A positive correlation between R_2_ and α can be appreciated by comparing both images, and was confirmed by linear fits of R_2_ to $sin^{4}\alpha$ . This was done separately for larger and smaller fibers with respect to the average diameter (4.46 μm), showing consistent angular dependence of R_2_ in both cases.

**Fig. 10.**
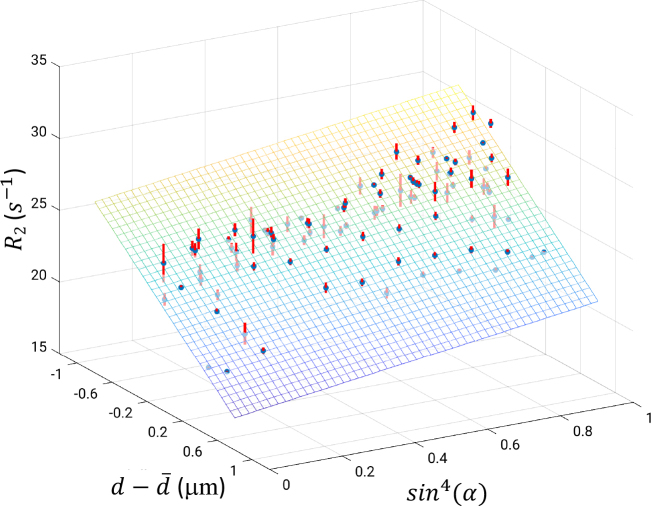
Surface fitting results for R_2_ against axon diameter index *d* and angle function $sin^{4}\alpha$ (bottom). Data are shown as mean (blue dots) ± standard error (red bars) of R_2_ for each fiber tract within $sin^{4}\alpha$ bins of 0.1 wide. Best fit in the least-squares sense was $R_{2}=22.08-6.06\;(d-\bar{d})+3.49sin^{4}\alpha$ ($s^{-1}$ ), $R^{2}=0.78$ .

With information about α and d, multi-linear regression of R_2_ to ($d-\bar{d})$ and $sin^{4}\alpha$ was performed and shown in Figure 10. An $R^{2}$ value of 0.78 was found, superior to linear regression with either ($d-\bar{d})$ or $sin^{4}\alpha$ alone (Table 1). Improvement with an additional term [($d-\bar{d})\cdot sin^{4}\alpha$ ] was marginal in terms of $R^{2}$, and was absent after adjustment for number of variables, suggesting negligible interaction by multiplication between the two explanatory terms in our data.

**Table 1.** Summary of fitting results using the reduced and full multi-linear model that accounts for fiber diameter and orientation.

	Coefficients for explanatory variables					
Fit No.	Intercept	$\left(d-\bar{d}\right)$	$sin^{4}\alpha$	$(d-\bar{d})\;\;\;\cdot \;\;sin^{4}\alpha$	*R^2^*	Adjusted *R^2^*
1	21.26	removed	5.34	removed	0.400	0.394
2	24.08	-7.33	removed	removed	0.632	0.628
3	22.08	-6.06	3.49	removed	0.784	0.780
4	21.96	-5.22	3.61	-1.57	0.787	0.780

## 4 Discussion

Using modified GESFIDE/GESSE acquisition and GESSE-style analysis, we performed whole-brain R_2_ mapping at 7 T MRI and observed sizable R_2_ variations of ~25% or 6 s^-1^ across the cortex, superficial white matter, and within deep white matter areas. In the cortical ribbon and superficial white matter, R_2_ closely followed putative tissue iron content and clearly reflected functional parcellation, with diminished influence from confounding factors such as the venous vasculature compared to R_2_^*^ and χ. Within deep white matter, R_2_ inversely correlated with fiber diameter and additionally correlated with fiber orientation. These observations are consistent with the expected manifestations of irreversible transverse relaxation considering its strong dependence on micro- and meso-scopic susceptibility as well as diffusion effects. Although these physiological contributions to brain R_2_ have been proposed and in some cases histologically validated (Balasubramanian et al., 2022; Hasan et al., 2012; Hervé et al., 2011; Kirilina et al., 2020; Yagishita et al., 1994; Zhou et al., 2001), this study offers a systematic investigation of their relative strengths in vivo at 7 T and their correspondences to whole-brain functional specialization.

The observation that the primary motor and visual cortices have much higher R_2_ than the emanating projection fibers indicates that R_2_ reflects iron content more strongly than myelination. This is also supported by the higher R_2_ in the superficial compared to the deep white matter, in keeping with the iron content distribution. Our findings are consistent with a recent study using high-resolution (250 μm) line-scanning GESSE at 7 T (Balasubramanian et al., 2022). The high resolution achieved in that study allowed detection of a central R_2_ peak across the cortical depth that closely follows non-heme iron distribution in contrast to myelin and microvasculature. A limitation of the study was the spatial coverage, which is now complemented by our whole-brain R_2_ results.

On the group analysis level, clear R_2_ differences between the cortex and underlying white matter were not observed in the primary somatosensory and auditory areas, in contrast to the primary motor and visual areas. This is partly attributed to a relatively high R_2_ in the white matter in somatosensory and auditory areas. For the primary somatosensory cortex, absence of observable R_2_ differences may also be attributable to the heterogeneous cellular and chemical compositions across subregions (BA 3a, 3b, 1 and 2) (Geyer et al., 1999), and increased partial volume effects due to reduced thickness compared to the primary motor cortex (1.81 mm vs. 2.69 mm as reported in Fischl & Dale, 2000). For the primary auditory cortex, literature is scarce to support the relatively high iron concentration seen in the other primary areas. If cortical R_2_ were indeed higher than the white matter, apparent differences may also be reduced by partial volume effects in this relatively thin cortical area, which is 2.34 mm thick on average according to Zoellner et al. (2019).

Based on our findings and relevant reports referenced above, we urge caution with using the ratio of T_1_-weighted and T_2_-weighted MRI data as indicator for cortical myelination at 7 T (Arshad et al., 2017; Glasser & Essen, 2011; Hagiwara et al., 2018), as the sensitivity of cortical R_2_ to iron may confound this interpretation. This may be especially problematic with iron accumulation associated with aging or neurodegeneration. To what extent iron confounds the interpretation depends on the acquisition parameters of the T_1_-weighted and T_2_-weighted images, yet it is expected to increase with the B_0_ field strength above 3 T as T_2_ is more sensitive than T_1_ to the increasingly stronger susceptibility effects from iron.

Because of its higher sensitivity to microscopic versus macroscopic susceptibility sources (Boxerman et al., 1995; Weisskoff et al., 1994), R_2_ may complement R_2_^*^ and χ (Cohen-Adad et al., 2012; Marques et al., 2017) to achieve higher specificity in high-field cyto- and myelo-architectural studies of the human cortex. Furthermore, R_2_ may be combined with R_2_^*^ and χ to separate paramagnetic and diamagnetic susceptibility sources that correspond to iron and myelin contributions respectively (Shin et al., 2021; Z. Li et al., 2023). Such separation may be enhanced by including T_1_ (R_1_) or quantitative magnetization transfer that are more specific to myelin (Marques et al., 2017; Sled, 2018; van der Weijden et al., 2021).

Iron concentration is known to increase with age in most of the brain (Hallgren & Sourander, 1958). Therefore, brain R_2_ is expected to generally increase with age as well, which has been demonstrated in selected brain structures (Balasubramanian et al., 2019; Luo & Collingwood, 2022). We expect the observed correspondence between R_2_ and cortical functional parcellation to remain with healthy aging, as the relative iron concentration of cortical areas roughly plateaus beyond the age of 40 years (Hallgren & Sourander, 1958). Nevertheless, other physiological and pathophysiological contributors to R_2_ through susceptibility or alternative mechanisms may modify this pattern, including demyelination, vascular changes, calcification, and amyloid deposition. Importantly, R_2_ is sensitive to the size of the underlying susceptibility sources, as observed in phantoms and demonstrated numerically to be related to diffusion distance (Weisskoff et al., 1994; van Gelderen et al., 2025). The notion is supported by the observation that sizable calcifications in the globus pallidus strongly change R_2_^*^ while only mildly changing R_2_ at 7 T (Balasubramanian et al., 2019). This again highlights the benefit of combining R_2_ with other susceptibility imaging methods, but also the caveats when interpreting them in tandem.

Another finding of our study was the negative correlation between white matter R_2_ and apparent axonal diameter, with an explanatory power of 0.63 by $R^{2}$ in linear fitting. Observation of white matter heterogeneity on T_2_-weighted MRI, particularly cortico-spinal tract (CST) hyperintensity, has been made early in the history of MRI (Curnes et al., 1988), and was attributed to the large fibers with thick myelination and wide translucent spaces in an joint MRI and histological study (Yagishita et al., 1994). Stanisz et al. discussed the interpretation of T_2_ relaxation in terms of water compartments and how these may be influenced by axonal geometry including fiber diameter (Stanisz et al., 2005). A previous quantitative T_2_ study at 7 T reported a 16% difference in T_2_ between the CST and its neighboring fiber tract (Hervé et al., 2011). The underlying mechanism of this effect likely is a complicated combination of the effects of diffusion (Kiselev & Novikov, 2018), susceptibility (Weisskoff et al., 1994), intercompartmental magnetization exchange (Dula et al., 2010), surface relaxation (Barakovic et al., 2023), and their interactions. It is noteworthy that a handful of physiological features share a similar brain topographical distribution as axonal diameter, including myelin thickness (Liewald et al., 2014), inter-axonal spacing (Kitajima et al., 1996), fiber orientation dispersion (Friedrich et al., 2020), and microvascular distribution and perfusion (Aslan et al., 2011). These features can also modify R_2_ to various extents. Separation of their contributions with current methodology is intractable due to the difficulty in manipulating them separately. Nevertheless, our results demonstrated that R_2_ can reflect these microstructural features two orders of magnitudes smaller than the voxel size, and therefore may complement diffusion MRI techniques for studying axonal diameter variations between major fiber bundles.

Research into noninvasive measurement of brain white matter fiber diameter using MRI has been a vibrant field, propelled mainly by advanced diffusion-weighted MRI techniques (Assaf et al., 2008; Fan et al., 2020; S. Y. Huang et al., 2020). This is a challenging endeavor, as the majority of human brain fibers have a diameter of less than 1 μm, necessitating extremely strong gradient hardware (S. Y. Huang et al., 2015) and meticulous modeling with consideration of complex tissue microstructures (Jelescu et al., 2020) for a faithful estimation of statistical parameters reflecting features of the underlying diameter distribution. Recent development using combination of relaxometry and diffusion may alleviate such demanding requirements and allow direct estimation of fiber diameters (Barakovic et al., 2023). A key step to their clinical applications is validation against histology, and cross-validation between in vivo methods, for which R_2_ mapping may be a viable candidate.

The orientation dependence of white matter transverse relaxation is a well-established effect, originating from the anisotropic magnetic susceptibility effect described by $\sin^{2}\alpha$ and/or $\sin^{4}\alpha$ terms (Oh et al., 2013; Wharton & Bowtell, 2012), and secondarily the magic-angle effect described by a ${\left(3\cos^{2}\alpha -1\right)}^{2}$ term (Bydder et al., 2007; Henkelman et al., 1994). In in vivo brain, such orientation dependence may be described using a single $\sin^{4}\alpha$ term (Bartels et al., 2022; Gil et al., 2016; Knight et al., 2017). The range of R_2_ orientation dependence observed in this study was 5.34 s^-1^, which is a factor of 2.43 compared to 2.2 s^-1^ at 3 T in Tax et al. (2021). This is close to the ratio of the field strengths 7/3 = 2.33, suggesting a close-to-linear scaling of R_2_ orientation dependence with B_0_. Intriguingly, based on R_2_ data from different head poses, Gil et al. (2016) reported that coefficients of the orientation-independent and dependent terms vary across fiber tracts, pointing to tract-specific R_2_ modifiers that were suggested to associate with fiber microstructures. This is in line with our findings of fiber diameter effects on orientation dependence. However, our data is limited to a single head pose in the supine position, which did not allow direct comparison with that study. In addition, using a multi-TE multi-shell diffusion spin echo EPI data acquired in two head-tilting positions (supine and 18° pitch), Tax et al. (2021) report that absolute R_2_ is higher and its orientation dependence much stronger for the extra-axonal signal than the intra-axonal signal. These findings together warrant further investigation of white matter R_2_ orientation dependence at 7 T by water compartmentalization and tissue microstructure.

Comparison of the orientation dependence of R_2_ and R_2_^*^ may elucidate their physiological origin. R_2_^*^ is known to be strongly orientation dependent primarily due to the diamagnetic susceptibility of myelin combined with water compartmentalization in white matter fibers (Duyn & Schenck, 2016). In the current study, we observed a R_2_^*^ variation of 5.60 s^-1^ across fiber tracts with the natural orientation dispersion in the supine position, similar to the value of 5.34 s^-1^ for R_2_. This similarity is in agreement with studies at 3 T, as Tax et al. (2021) report R_2_ variation of 2.2 s^-1^, and Bender and Klose (2010) report R_2_^*^ variation of 2.68 s^-1^, both pooling whole-brain white matter voxels. However, the strength of orientation dependence varies across fiber tracts, particularly for R_2_ (Gil et al., 2016; Tax et al., 2021), which may reflect the tract differences in microstructure such as fiber diameter and myelin thickness. Therefore, studies of orientation dependence of single fiber tracts with multi-orientational data would be more revealing. For example, in in vivo marmoset brain at 7 T, a 15 s^-1^ increase in R_2_^*^ was found in the optic radiation when the fiber tract was perpendicular versus parallel to the B_0_ (Sati et al., 2012).

A confounding factor with our fiber-tract specific R_2_ analysis is the variation in fiber diameter and orientation within regions of interest (Jeurissen et al., 2019). Notably, orientation dispersion of fine fibers itself might not strongly affect R_2_ of the voxel through susceptibility effects as measured by GESSE in this study, because of the diffusion averaging effect associated with the long TE of 40 ms (van Gelderen et al., 2025; Yablonskiy & Haacke, 1997). The practical effect of multiple fiber orientations and/or fiber diameters within a voxel is a “noisy” appearance of the R_2_ distribution within white matter (Bender & Klose, 2010; Gil et al., 2016; Tax et al., 2021). We alleviated this effect by limiting the analysis to voxels with an FA larger than 0.4 and tract probability higher than 0.25, effectively selecting voxels dominated by a single tract. Such approach can be further enhanced by measuring subject-specific diffusion images to avoid misregistration to standard templates. This will also open new opportunities for joint analysis of R_2_ with advanced diffusion-derived biomarkers such as neurite density and orientation dispersion index (H. Zhang et al., 2012), non-Gaussian diffusion (Steven et al., 2014), axonal diameter distributions (Assaf et al., 2008), compartment-specific parameters (Gong et al., 2020), among others.

Decades of relaxometry research have demonstrated that irreversible transverse relaxation in a complex environment such as biological tissues is dependent on a myriad of effects (e.g., nature of interaction of water with other molecules, water diffusion, magnetization exchange, and magnetic susceptibility) and their interactions, rendering it non-exponential and TE-dependent in nature. Therefore, T_2_ values obtained through quantitative MRI heavily depend on the method of measurement and should be considered “apparent” T_2_ that partially reflects the underlying irreversible transverse relaxation. For example, multi-echo spin echo in the CPMG regime is well-established to reduce effects of tissue intrinsic diffusion as are encountered in Hahn T_2_ (Carr & Purcell, 1954); However, diffusion weighting from the imaging gradients becomes significant when imaging at high spatial resolution (Oakden & Stanisz, 2014) or in the presence of imperfect refocusing as a result of B_1_ heterogeneity (Weigel & Hennig, 2012). GESSE T_2_ has similar diffusion contributions as Hahn T_2_ (van Gelderen et al., 2025), yet discrepancies may arise when TEs of Hahn SEs are different from that of GESSE due to multi-compartmental relaxation in tissues.

To estimate R_2_, we followed the original GESSE method (Yablonskiy & Haacke, 1997) that took the ratio of symmetric echo pairs around the spin echo. This model-free approach is insensitive to the underlying frequency distribution, which may deviate from Lorentzian. For example, in some brain regions at high field, Gaussian distributions were found to be superior to Lorentzian (Mulkern et al., 2015). A flexible distribution model may improve quality of fitting when both irreversible and reversible transverse relaxation characteristics are of interest (Steidle & Schick, 2021).

The T_2_ data presented here were acquired in 1 × 1 × 2 mm^3^ resolution, which is rather modest to state-of-the-art T_2_^*^ acquisitions commonly performed at 7 T. When using a large (3 mm) slice separation as done here, misregistration and partial volume effects can compromise accuracy of R_2_ on the cortical and SWM surfaces. We attempted to minimize the adverse effects by using high-resolution T1MPRAGE for cortical parcellation, supplying a T_2_(T_2_^*^)-weighted image to “bbregister” with the “-t2” option enabled, and checking individual alignment. Higher resolution R_2_ data would allow more accurate study of the dependence of R_2_ on cortical area and depth, but would require further technical development to obtain substantial volume coverage within a practical scan duration. This could be addressed by imaging acceleration using parallel imaging and multi-shot EPI methods (Uğurbil et al., 2013; Z. Zhang et al., 2022; Y. Huang et al., 2024). Combination with low-rank signal modelling for continuously acquired GE trains may further improve scanning efficiency (Cao et al., 2022; Wang et al., 2022) to allow higher resolution. Caution needs to be taken as effects such as magnetization transfer, intercompartmental exchange and refocusing RF efficiency can impact the observed R_2_. Such effects need to be considered in pulse sequence design or modeled in post-processing to achieve higher consistency and reproducibility. An alternative approach for acceleration of the image acquisition would be multiband or simultaneous multislice imaging (Larkman et al., 2001). One caveat would be the increased RF power deposition and maximum RF amplitude, which may be alleviated by further reducing pulse bandwidth, lowering flip angle, or using PINS pulses (Norris et al., 2014). Research efforts in these directions are warranted considering the unique value afforded by brain R_2_ in 7 T studies of healthy aging and neurodegenerative diseases.

## 5 Conclusion

Whole-brain R_2_ maps can be robustly acquired at 7 T with the GESSE approach and show dominating contributions of iron in both cortical and deep gray matter regions. Robust against the confounding contrast from venous vasculature characteristic of susceptibility-weighted techniques, R_2_ may contribute to assessing gray matter iron content. In major white matter fiber bundles, both fiber diameter and orientation relative to B_0_ show an apparent contribution to R_2_, complicating its interpretation.

## Supplementary Material

Supplementary Material

## Data Availability

The underlying data and code that support the findings of the study are available from the corresponding author upon reasonable request, and subject to institutional policies.
