# Supplementary material for: T2 contrast variation in human brain at 7 T and its potential contributors
Source: Imaging Neurosci (Camb). 2025 Jul 7;3:IMAG.a.67. doi: 10.1162/IMAG.a.67 (PMC12330860; doi:10.1162/IMAG.a.67)
Supplement: Supplementary Material [file IMAG.a.67_supp.pdf]

SUPPLEMENTARY MATERIAL

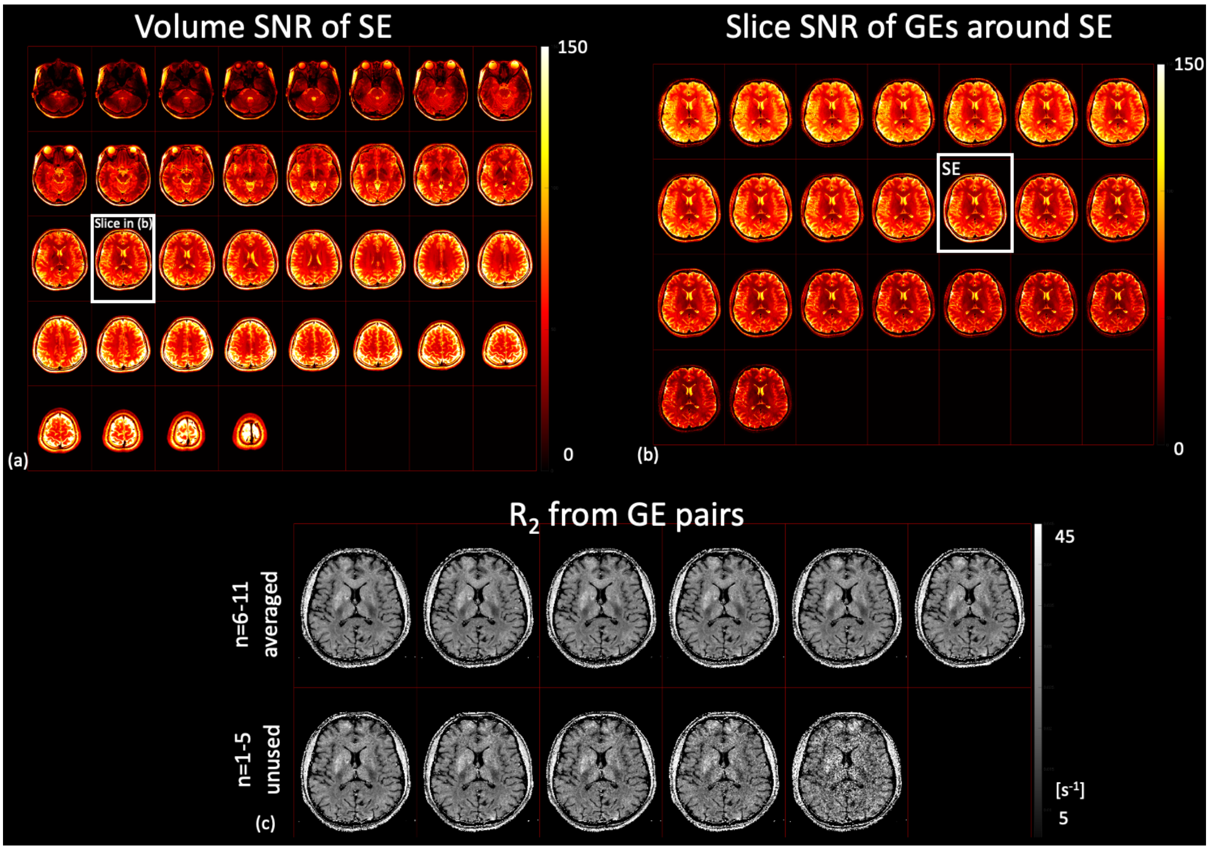

Figure S1. Volume SNR maps for the spin echo (a), SNR maps for the gradient echo around the spin echo (b), and calculated R_2 from each gradient echo pairs.

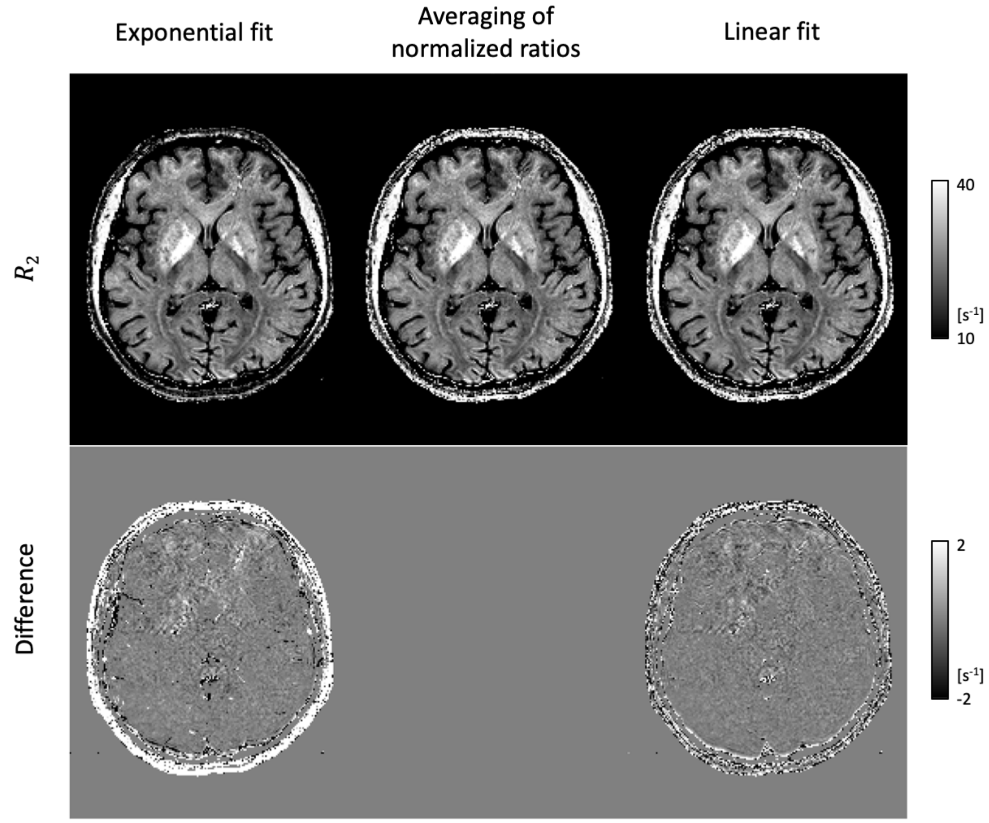

Figure S2. Comparison of methods for R_2 estimation. The exponential fit included 3 independent variables M_0, R_2, R_2'. The linear fit found R_2 as the slope of log signal ratio of GE pairs versus $2n\Delta TE$. These two methods led to similar results as that used in the main study.

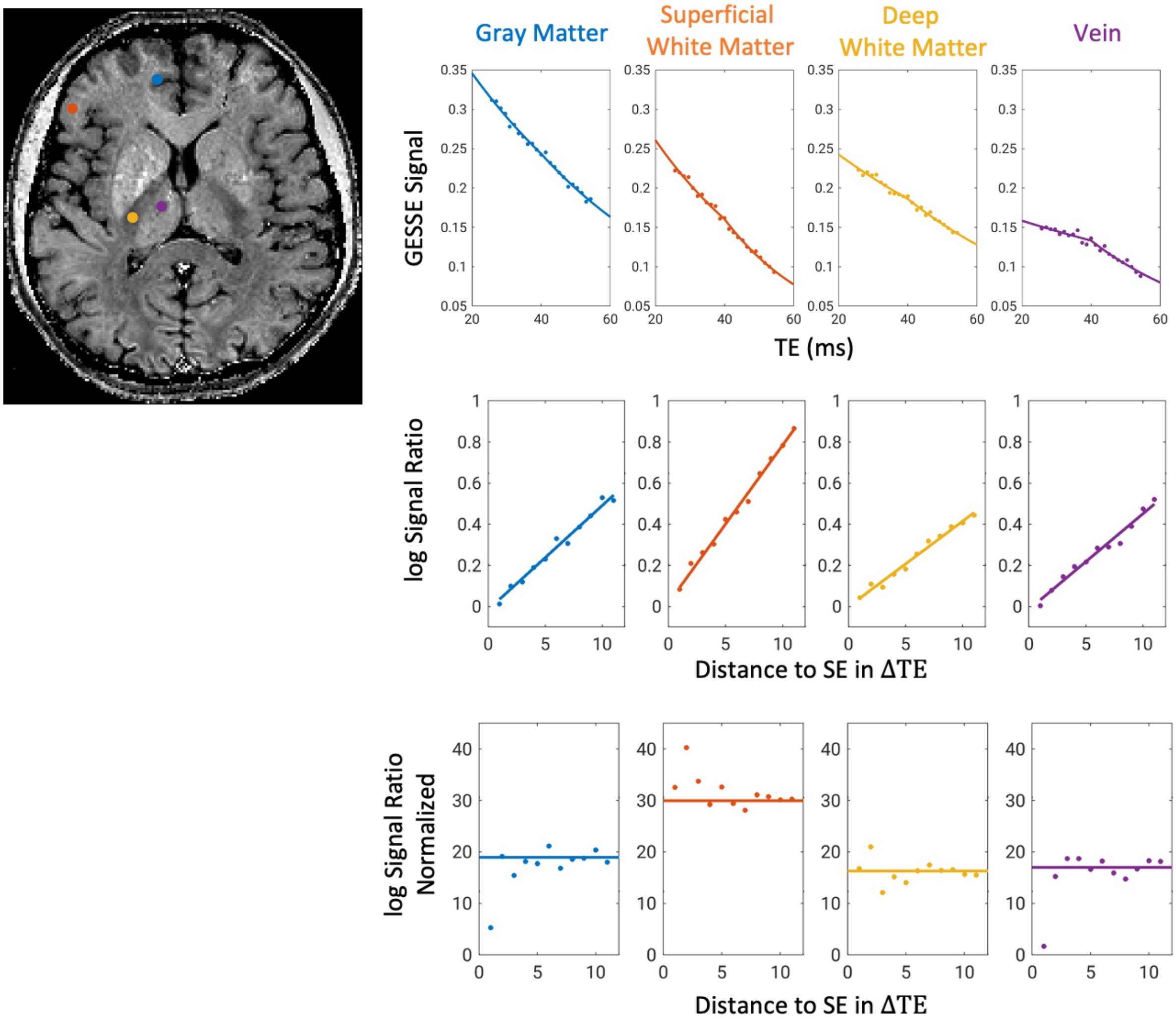

Figure S3. Example fits for voxels belonging to 4 different tissues. Top row is exponential fit to GESSE signals, middle row is linear fit to log signal ratio of GE pairs, and bottom row shows normalized ratios (i.e. R_2 in ms) and the average from n=6-11.

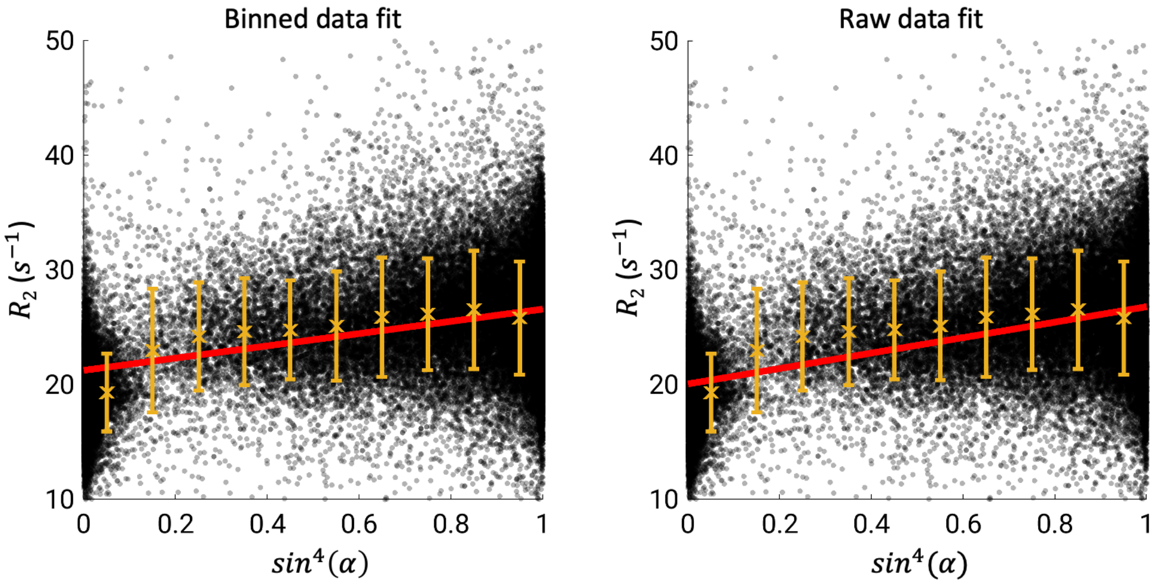

Figure S4. Raw R_2 data and fiber orientation in the white matter of 12 subjects. Yellow error bars show mean±standard deviation of the binned data by $sin^4\alpha$. Red lines are the linear fit to the binned data on the left, and that to the raw data on the right.

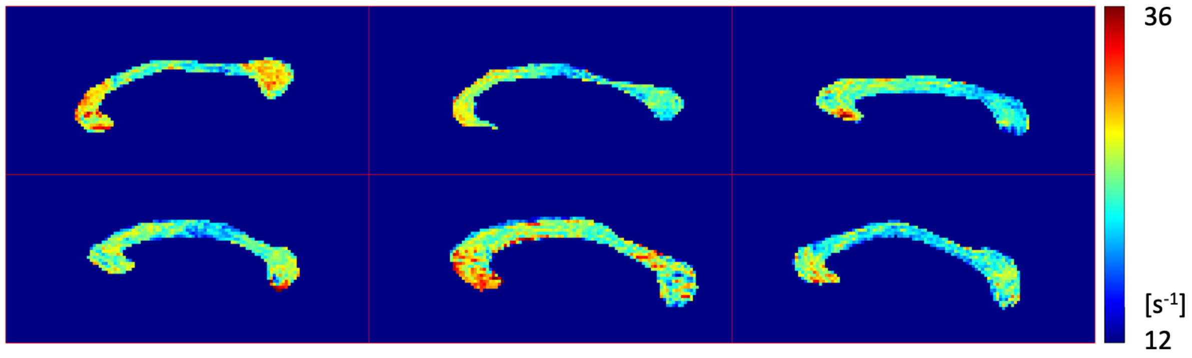

Figure S5. Corpus callosum R_2 on the mid-sagittal plane from 6 subjects.
